# Fabrication of Silicon Nanowire Sensors for Highly Sensitive pH and DNA Hybridization Detection

**DOI:** 10.3390/nano12152652

**Published:** 2022-08-02

**Authors:** Siti Fatimah Abd Rahman, Nor Azah Yusof, Mohd Khairuddin Md Arshad, Uda Hashim, Mohammad Nuzaihan Md Nor, Mohd Nizar Hamidon

**Affiliations:** 1 Institute of Nano Electronic Engineering, Universiti Malaysia Perlis, Kangar 01000, Perlis, Malaysia; mohd.khairuddin@unimap.edu.my (M.K.M.A.); uda@unimap.edu.my (U.H.); m.nuzaihan@unimap.edu.my (M.N.M.N.); 2 Institute of Nanoscience and Nanotechnology, Universiti Putra Malaysia, Serdang 43400, Selangor, Malaysia; mnh@upm.edu.my; 3 Department of Chemistry, Faculty of Science, Universiti Putra Malaysia, Serdang 43400, Selangor, Malaysia; 4 Faculty of Electronic Engineering Technology, Universiti Malaysia Perlis, Arau 02600, Perlis, Malaysia

**Keywords:** silicon nanowire, biosensor, pH, DNA hybridization, electrical detection

## Abstract

A highly sensitive silicon nanowire (SiNW)-based sensor device was developed using electron beam lithography integrated with complementary metal oxide semiconductor (CMOS) technology. The top-down fabrication approach enables the rapid fabrication of device miniaturization with uniform and strictly controlled geometric and surface properties. This study demonstrates that SiNW devices are well-aligned with different widths and numbers for pH sensing. The device consists of a single nanowire with 60 nm width, exhibiting an ideal pH responsivity (18.26 × 10^6^ Ω/pH), with a good linear relation between the electrical response and a pH level range of 4–10. The optimized SiNW device is employed to detect specific single-stranded deoxyribonucleic acid (ssDNA) molecules. To use the sensing area, the sensor surface was chemically modified using (3-aminopropyl) triethoxysilane and glutaraldehyde, yielding covalently linked nanowire ssDNA adducts. Detection of hybridized DNA works by detecting the changes in the electrical current of the ssDNA-functionalized SiNW sensor, interacting with the targeted ssDNA in a label-free way. The developed biosensor shows selectivity for the complementary target ssDNA with linear detection ranging from 1.0 × 10^−12^ M to 1.0 × 10^−7^ M and an attained detection limit of 4.131 × 10^−13^ M. This indicates that the use of SiNW devices is a promising approach for the applications of ion detection and biomolecules sensing and could serve as a novel biosensor for future biomedical diagnosis.

## 1. Introduction

Biosensors have undergone significant advancement since their inception in 1962 [1,2]. Genetic information obtained via genome sequencing has significantly contributed to the development of deoxyribonucleic acid (DNA) biosensors. Owing to their immobilized DNA strands, DNA biosensors can detect complementary sequences via hybridization. Several detection methods, including optical, electrochemical, electrical, and mass-based devices, can be used to determine hybridization between a probe and its complementary sequence [3,4]. Some of these detections require labels, such as fluorophores, organometallic complexes, or redox indicators, to attach to the DNA target. Although labeling improves sensor sensitivity, labeling-based detection increases the time, complexity, and cost of measurement.

Huge efforts have been made to develop an electrical-based biosensor that offers a simple, rapid, label-free, low-cost detection of DNA hybridization with direct electrical readout [5,6,7]. The use of electrical biosensors began in 1997 through the development of a field-effect transistor device for DNA hybridization detection. Despite its label-free feature, operational dimensions of biosensors were in the millimeter range, with recording buffer concentrations in the order of a millimole [8]. Thus, integrating nanomaterials into device structures for biosensing applications is crucial for developing new strategies for signal transduction. Synergizing nanomaterials and biomolecules can help develop miniaturized sensing devices for detecting biomolecules because biological molecules and nanomaterials have comparable sizes [9,10,11], making nanotechnology a strategic approach for surpassing standard sensor limits.

Various nanoscale materials, such as nanowires [12], nanotubes [13], and nanoparticles (including quantum dot) [14,15], exhibit excellent prospects for biosensing applications. In particular, silicon nanowire (SiNW) is a very well-defined and controlled class of nanoscale building blocks, owing to its adaptability for semiconductor-based technology. Furthermore, silicon (Si) is widely used in biosensor development due to its biocompatibility with semiconducting materials. SiNW is used in the standard configuration of field–effect devices as a semiconducting channel (active layer of the device) that is connected between the source and drain electrodes. The current is injected form the metal source, subsequently draining the electrodes through which the current is collected. The presence of charged analytes on the nanowire surface will result in a change in the charge density of opposite sign in the SiNW space-charge region. These field-effect sensors can detect various biomolecules and chemical species based on nanowire surface modification with specific receptors [12,16,17].

Furthermore, the intrinsic silicon dioxide (SiO_2_) surface of SiNW can be simply functionalized with various probe molecules, thereby rendering SiNW a direct and specific sensor [18,19]. Most reported methods used single-stranded DNA (ssDNA) probes as receptors to hybridize the target DNA. Owing to their simple synthesis, ease of labeling, and high stability, ssDNA molecules have become ideal recognition probes for DNA hybridization detection [20,21,22]. Prior to biomolecules attachment, the SiO_2_/Si surface is modified using 3-aminopropyltriethoxysilane (APTES) and glutaraldehyde to provide a linking site between nanowire surfaces and the recognition group of the DNA molecules [19,23]. 

Reports have shown that bottom-up grown SiNW using the well-established chemical vapor deposition technique have poor control of nanowire diameter and are difficult to precisely position to other existing microelectronic components [24,25]. These issues have been resolved with the introduction of SiNW devices patterned by top-down lithography [12,26]. This method involves transferring a geometric pattern from a mask to a chemical resist on a planar surface, followed by the continuous application of various etching techniques. This approach can yield well-aligned SiNW, which are highly uniform, reproducible, and easily integrable into electrical readout circuits [27]. 

This study presents a top-down fabrication method for device patterning using electron beam lithography (EBL) combined with the standard complementary metal–oxide–semiconductor (CMOS) process. Investigations of different diameters and different numbers of SiNWs in response to different pH levels are performed to study the effect of surface charges, which induce a depletion or accumulation region in SiNWs, thereby resulting in a more significant effect on device sensitivity. To evaluate the performance of the developed SiNW sensor in DNA hybridization detection, dengue virus (DENV) DNA is selected as the detection target. This study demonstrates the potential of SiNW as a sensing platform for detecting specific target analytes.

## 2. Materials and Methods

### 2.1. Materials

A 4-inch p-type silicon on insulator (SOI) wafer (Soitec, Bernin, France) with a top Si layer thickness of 500 Å, a buried oxide layer thickness of 2000 Å, and supported by 500 µm thick Si substrate was used as a starting material for the device fabrication. Chemical used for surface modification such as phosphate buffer saline (PBS, pH 7.4), (3-aminopropyl)triethoxysilane (99%, APTES), and glutaraldehyde solution (~25% in PBS, GA) were obtained from Sigma-Aldrich (St. Louis, MO, USA). Commercial pH buffer solutions (i.e., pH 4, pH 7, and pH 10) were purchased from QREC (Selangor, Malaysia) and used without further modification. The 27-mer single-stranded DNA (ss-DNA) probe, its complementary oligonucleotide, non-complementary oligonucleotide, and one-base mismatched oligonucleotide used in this work were 5′−NH_2_−C_6_−AAC AGC ATA TTG ACG CTG GGA GAG ACC−3′, 5′−TGG CTC CCT GAC CGT CAA TAT GCT GTT−3′, 5′−CTT TGT GTT AGT ATC TGG CCG ATG TCC−3′, and 5′−GGT CTT TCC CAG CGT CAA TAT GCT GTT−3′, respectively. All the oligonucleotide sequences were synthesized by Integrated DNA Technologies (Coralville, IA, USA). and stored at −20 °C. 

### 2.2. Mask Design

**Figure 1:**
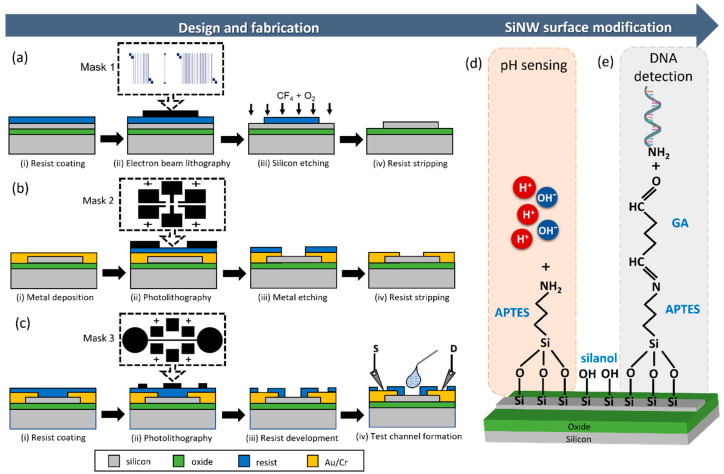
A schematic diagram of top-down fabricated SiNW sensors using EBL mixed photolithography-based CMOS processes with (**a**) Mask 1 for nanowire pattering, (**b**) Mask 2 for fabricating electrode pads, and (**c**) Mask 3 for developing the test channel. (**d**) Chemically modified SiNW using APTES for pH sensing. (**e**) Further surface modification using glutaraldehyde to form GA/APTES/SiNW for amine-terminated ssDNA probe immobilization.

Nanowires (NWs) patterns were created before the fabrication process using a computer-aided design program developed by Raith GmbH, which is known as Elphy Quantum GDS II Editor that is attached with electron beam lithography (EBL) system. An overview of the Mask 1 layout design, which includes a set of three different numbers of NWs (10 NWs array, single NW, and 20 NWs array), is illustrated in Figure 1a. Notably, each set of NWs was designed in various diameter scales ranging from 60 to 100 nm. In creating these mask layouts, certain design constraints were considered. A long nanowire with a dimension of 400 µm was designed to ensure that the structure is in contact and overlaps with the electrode pad in the forthcoming fabrication process. The NW arrays were then separated by 20 µm gaps to allow for future development and etching capabilities. Furthermore, micrometer-sized square structures were designed at the corners of each NW array and at the end of the single NW to serve as alignment keys for the forthcoming photolithography fabrication process.

Then, two designs, which are electrode pad (Mask 2) and test channel (Mask 3) were designed using AutoCAD software. Mask 2 was used to create the electrode pads of the SiNW devices (Figure 1b). The pads were designed on a millimeter scale to provide enough space for placing the probing point during the electrical measurement stage. Additionally, the source and drain pads are separated by 0.250 mm to provide a space for NW placement. According to the designs, each array of the NW structures will have its set of electrode pads and will be formed as three distinct devices. On the SiNWs device (Figure 1c), Mask 3 was designed to define the open region for contact pads and the analyte dropping area. The dimensions of the microfluidic channel were 6 mm in length and 0.15 mm in width, with 3 mm diameter inlet/outlet holes. The design allows for the simultaneous measurement of three devices in parallel, significantly increasing the amount of data for a given experiment. Finally, the AutoCAD design was printed onto a 5-inch square chrome glass surface as a photomask for the photolithography process. 

### 2.3. Fabrication of Silicon Nanowire Sensor 

In the device fabrication, a top-down approach based on an EBL system (ELS-7000, Elionix, Tokyo, Japan) coupled with conventional photolithography (MIDAS MDA-400M Mask Aligner, Daejeon, Korea) was used. The entire fabrication process was conducted based on standard CMOS fabrication procedures. The process flow used to create SiNW on SOI sample is illustrated in Figure 1a. In the first step, the cleaned sample was coated with e-beam resist ma-N 2403 (Microresist Technology GmBH, Berlin, Germany). The sample was then baked on a hot plate at 180 °C for 2 min and cooled down to room temperature. During the EBL process, the NW design (Mask 1) was directly exposed to the coated resist sample using a beam of electrons with a beam voltage of 20 kV. The developed resist pattern was then transferred into the top Si layer of the sample using inductively coupled plasma reactive ion etching (ICP-RIE). The etchant gases of CF_4_ (30 sccm) and O_2_ (28 sccm) were used to etch the area that was not covered by the resist mask. The resist mask was removed with acetone, revealing SiNWs with a good anisotropic profile. 

A thin layer of 500 Å gold (Au) and 300 Å chromium (Au/Cr) was then deposited onto the fabricated SiNW sample, followed by patterning the electrode pads design (Mask 2) with conventional photolithography on the coated PR1-2000A positive resist sample. The developed resist acts as a hard mask for subsequent etching steps using aqua regia solution (3:1 hydrochloric acid (HCL); nitic acid (HNO_3_)) at room temperature, as shown in Step (iii) in Figure 1b. The resist layer was then stripped using acetone, and the desired Au/Cr pattern was performed at the end of both sides of the SiNWs to form the source (S) and drain (D) contacts. Following that, the test channel was lithographically patterned using Mask 3 to develop an opening window on the SiNWs detection region and metal pads (Figure 1c). Before testing the aqueous solution, the SU-8 resist layer was introduced as a passivation layer. Briefly, the device was coated with the SU-8 negative resist before being baked at 100 °C for 90 s. The coated sample was photolithographically exposed to UV light and then immersed in RD6 developer for 30 s. During the development process, the exposed area of negative resists through UV radiation remained, resulting in the opening windows on the desired areas. Finally, the sample was baked using a hot plate at 100 °C for 3 min and cooled down to room temperature. The purpose of forming a microfluidic channel is to provide precision in the delivery of analytes in small volumes while acting as a passivation layer to protect the electrode pads from short-circuiting the sensing current.

### 2.4. Surface Modification and Detection

#### 2.4.1. pH Sensing

To evaluate the effectiveness of the fabricated SiNW device as a sensing tool, a preliminary test was performed by demonstrating the device for the case of pH sensing. For this purpose, the fabricated SiNW sensor was functionalized with APTES, which was prepared by mixing 2% APTES in a mixture of ethanol and deionized water (95%/5%, *v*/*v*) for 2 h at room temperature. The samples were rinsed using ethanol and deionized water (DIW) and subsequently dried on a hot plate at 120 °C for 30 min. These modification steps provide a surface terminating with amine groups that can undergo protonation or deprotonation with pH solution (Figure 1d). The characterization was started with 10 µL of pH 4, followed by pH 7 and pH 10. Between solution exchanges, the samples were rinsed with DIW and blow-dried before introducing a new pH solution. The responses of the device were recorded based on the change of the current–voltage (IV) measurement. 

#### 2.4.2. DNA Hybridization Detection 

Figure 1e shows the coupling of an amino group ended single-stranded DNA (NH_2_-ssDNA) probe to an aldehyde-terminated sensing surface. For ssDNA immobilization, a similar procedure was used for the silanization process of SiNWs with APTES, followed by GA modification steps. The one-end of bi-functional aldehyde groups (CHO-(CH_2_)3-CHO) in GA can link with the amine groups of APTES/SiNW to promote the termination of GA/APTES/SiNW on the other end of the aldehyde group for further attachment with NH_2_-ssDNA probe [23]. Briefly, the APTES/SiNW samples were placed in GA solution for 1 h at room temperature. The samples were then rinsed with PBS solution and dried with blowing air. The GA/APTES/SiNW were then incubated in 10 µL of 1 µM of ssDNA probe (diluted using PBS, pH 7.4) overnight. After PBS washing to remove the non-bound ssDNA probe, IV measurement was conducted to determine the ssDNA/GA/APTES/SiNW. 

For the hybridization process, 10 µL of complementary target DNA applied onto the ssDNA/GA/APTES/SiNW for 2 h at room temperature. Following that, the unbound target DNA was removed by washing the sample with PBS buffer and subsequently blow-dried. A similar procedure was used for the hybridization of non-complementary target and mismatch target DNA sequence on the ssDNA/GA/APTES/SiNW. The electrical measurement was used to observe the electrical response of different target DNA hybridized on the ssDNA/GA/APTES/SiNW.

### 2.5. Characterization 

Electrical characterization was performed using a Keithly 6487 Picoammeter probe station. A typical resistor setting was used on the two-terminal NW devices by supplying sweep voltages of 2V to the source region to obtain an output current at the drain region. The measurement was done inside a dark shielded cage to isolate it from electromagnetic noise and optical illumination. High-power microscope (HPM) (Olympus BX 51, Olympus Corporation, Tokyo, Japan), X-ray photoelectron spectrometer (XPS) (Axis Ultra DLD, Shimadzu Corporation, Kanagawa, Japan), profiler surface analysis (Hawk 3D WT-250, Pemtron, Seoul, Korea), and field-emission scanning electron microscope in combination with energy-dispersive X-ray (FESEM/EDX-Hitachi H7100, Hitachi Ltd, Tokyo, Japan) were used for surface and morphological characterization.

## 3. Results and Discussion

### 3.1. Top-Down Fabricated SiNW Device

**Figure 2:**
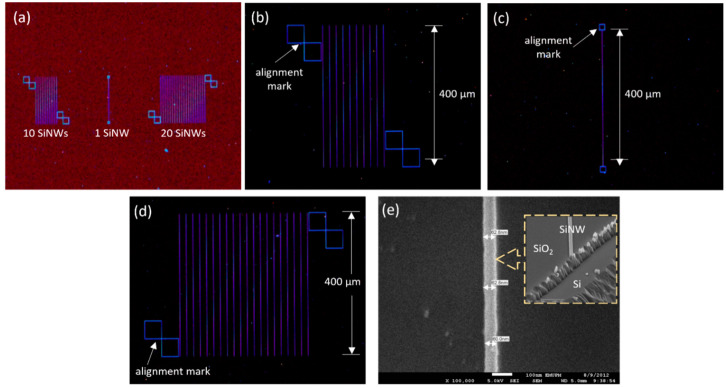
(**a**) HPM images of fabricated SiNWs and zoom image of (**b**) 10 SiNWs, (**c**) single SiNW, and (**d**) 20 SiNWs. (**e**) Top view of 60 nm width of SiNW structure. The inset shows a cross-section of the SiNW fabricated on the SOI sample.

HPM with magnifications of 5× and 20× were used to inspect the overall pattern developed on the device. The dark-field image in Figure 2a shows that the designed nanowire (Mask 1), which consists of a single and array format, are well-replicated onto the sample substrate by the EBL and plasma-etching process. Figure 2b–d illustrated the individual images of SiNWs structures, with the blue color denoting the developed structure of SiNWs on the SiO_2_ surface. Three sections of NWs with different numbers were fabricated in parallel with 400 µm in length. A group of 10 NWs and 20 NWs were developed with a set of two adjoining square shapes, respectively, while for a single NW, a set of square shapes was developed at the end of the structure as well. This micro-sized of the square shapes was designed as the alignment marker for the forthcoming electrode pad fabrication using the conventional photolithography process. Since the nanoscale structures are difficult to be seen with the naked eye, the markers play an important feature for placing the SiNWs structures at the accurate coordinate in this separate patterning of the millimeter-sized contact pads.

Additionally, to assess the size effect of the SiNWs on the electrical properties, samples with different widths of the SiNWs were fabricated using EBL, by varying the design width of the NW pattern for each exposure. In this context, the number and the length of NWs were fixed, while the width was varied, i.e., 60–100 nm from die to die. After the Si etching process, the corresponding results were characterized using an FESEM image, as shown in Figure S1 of Supplementary Materials. Furthermore, the SOI-based top-down approach produces a rectangular cross-section of nanowire, as shown in the inset Figure 2e. The fabricated SiNW has a height of approximately 50 nm, which corresponds to the top Si layer thickness of the SOI wafer as the starting material. This shows the successful fabrication of horizontal siNWs with good quality.

**Figure 3:**
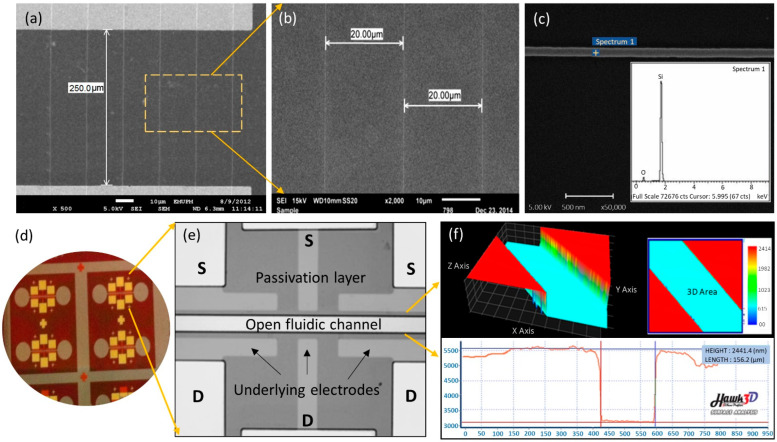
FESEM images of (**a**) SiNWs bridged a 250 µm gap of electrodes and (**b**) a 20 µm gap between SiNWs. (**c**) EDX analysis of nanowire surface. (**d**–**f**) The developed pattern of Mask 3 as a final device layout.

Once the SiNWs have been fabricated, the subsequent step was to form the electrode pad via the conventional photolithography process. Typical FESEM images of the SiNWs arrays are illustrated in Figure 3, showing that the source and drain contact pads are well aligned on top of the individual NWs with 20 µm spacing between the wires. Thereafter, the elemental analysis of the developed SiNWs was determined by the energy-dispersive X-ray (EDX) equipped with FESEM. A peak of silicon (Si) and oxygen (O) with total percentage weights of 77.8% and 22.2%, respectively, were observed on the spotted SiNW, as illustrated in Figure 3c. This thin oxide layer can be chemically modified with APTES and GA, transforming the fabricated SiNWs structure into a functional (bio)sensor device [23]. Figure 3d–f depicts the final view of the device fabricated using Mask 3. The opening windows on the source (S) and drain (D) of the electrode pads as well as the SiNWs channel region are formed well, as observed by the HPM image. The fluidic channels are well-developed with a gap of approximately 150 µm and a passivation layer thickness of approximately 2.5 µm, as illustrated in a three-dimensional view using a surface-profiler image (Figure 3f). 

### 3.2. Surface Analysis of Modified SiNW

**Figure 4:**
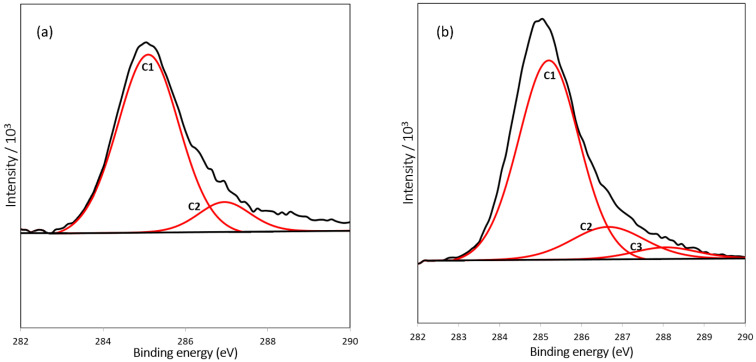
XPS spectra of C1s for Si surface modified with (**a**) APTES and (**b**) glutaraldehyde.

XPS was used to characterize the resulting layers on the silicon surface after modification with APTES (APTES/SiNW) and glutaraldehyde (GA/APTES/SiNW). Bond types formed after chemically modifying the Si surface can be detected using XPS [28]. The C1s orbital signal of the carbon element can be observed at 285 eV. The fitting of the C1s spectra revealed several species on the sample surface (Figure 4). In addition to the hydrocarbon species (C1) that indicated adventitious surface contamination, the presence of the second component (C2) attributed to the C–N and C–O bonds validates the amine functionalization of SiNW using APTES. Furthermore, a new species (C3) that appeared at the peak of 288.05 eV signifies aldehyde group formation (Figure 4b). The highly oxygenated carbon species (C=O and O–C–O) confirmed the effective surface functionality of glutaraldehyde [28,29]. Table 1 lists the binding energy and percentage area of the elements from the C1s spectra.

### 3.3. Optimization of SiNW Sensors

For the development of the SiNW sensors, a few studies reported that the sensitivity of the sensor devices could be affected by several factors including the design specification of SiNWs (e.g., nanowire width and nanowire number) [30,31,32]. Thus, the optimization of fabricated devices was first performed according to the pH response. 

#### 3.3.1. Effect of Different Widths of SiNW on Device Sensitivity

**Figure 5:**
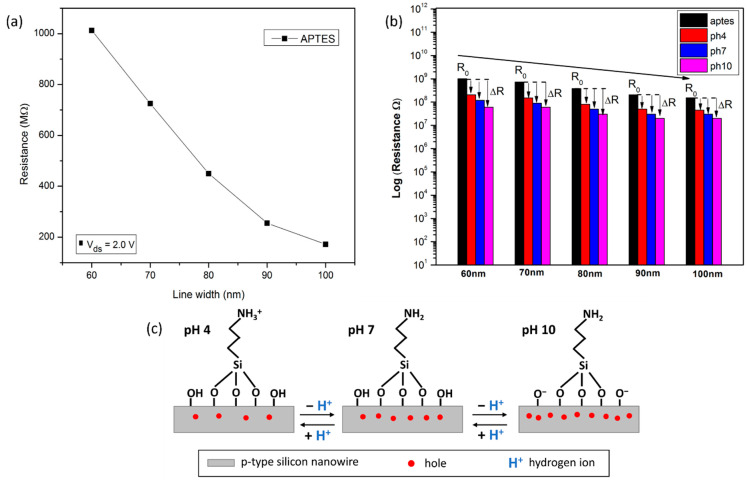
(**a**) The average resistance measurement of sensor devices dependent on the width of SiNW in response to the APTES modified. (**b**) The current response of fabricated devices with different widths of SiNW for pH level detection (i.e., pH 4, 7, and 10). (**c**) The underlying mechanism of the p-type SiNW for pH sensing.

The fabricated sensor devices consisting of a single NW with varying widths from 60 to 100 nm (refer to Figure S1 of Supplementary Materials) were further electrically characterized in response to the different pH solutions. This feature began by adding a drop of 10 µL solution of pH 4, and then increased step-wisely to pH 7 and 10. In between the pH solution change, the devices were rinsed with DIW, and the signal dropped back to APTES/SiNW signal, which is the baseline of this measurement. As shown by the data points in Figure 5a, the device’s resistivity values were related to the width of the Si wire. The average resistance of the fabricate devices at V_ds_ = 2V with wire widths of approximately 60, 70, 80, 90, and 100 nm were 1013 × 10^6^ Ω, 725 × 10^6^ Ω, 450 × 10^6^ Ω, 255 × 10^6^ Ω, and 172 × 10^6^ Ω, respectively. The resistance of the wire was found to be inversely proportional to its width, which is consistent with previous reports [33,34].

The electrical response of pH sensing associated with different widths of SiNW, as obtained in Figure S2 of Supplementary Materials, is summarized in Figure 5b, in logarithmic scales. It clearly shows that the samples of different widths of SiNW exhibited a similar decreasing trend of resistance responses upon increasing the pH from 4 to 10. The SiNW pH response was evaluated via the interaction between surface silanol (SiOH) group and amino (NH_2_) group undergoing protonation and deprotonation as the hydrogen ion (H^+^) concentration fluctuated (Figure 5c). Due to the high H^+^ ion concentration in acidic solution (pH less than 7), the NH_2_ group is protonated to NH_3_^+^ (NH_2_ + H^+^ ⟺ NH_3_^+^). Thus, more positive surface charge is generated and results in depletion of charge carriers (holes) in the p-type SiNW, leading to increase in the resistance for pH 4. Conversely, for basic solution (pH greater than 7), the SiOH group becomes deprotonated to SiO^−^ (SiOH ⟺ SiO^−^ + H^+^). Subsequently, this generates more negative surface charge on the nanowire surface, which depletes the hole carries and decreases the resistance for pH 10. This underlying concept was first proposed by Cui et. al [35] for the case of a pH sensor. 

The principal operation of SiNW sensor suggests that when the analytes bind the receptors, the additional field effect charges close to the surface induce opposite charges within the semiconductor channel, which is then detected as a change in current (resistance or conductance). Thus, the sensor response for pH measurement can be defined as the relative change in resistance (R) and can be expressed as:

∆R/R_0_ = (R_pH_ − R_0_)/R_0_, (1)

where R_0_ is the resistance of APTES/SiNW (baseline), and ∆R is the change in resistance after introducing the pH solution [33]. At V_ds_ = 2V, the sensor consisting of SiNW with varying widths of 60, 70, 80, 90, and 100 nm demonstrated a device response to pH 4 of approximately 74%, 63%, 54%, 40%, and 23%, respectively. It was found that increasing the width of the NW reduces the device response towards different pH levels (i.e., pH 7 and pH 10), as shown in Table S1 of Supplementary Materials. In addition, the sensors that consist of the smallest width of SiNW, with 60 nm, show the highest sensitivity approximately 18.26 × 10^6^ Ω/pH, while the lowest sensitivity, approximately 3.34 × 10^6^ Ω/pH, is observed by using a 100 nm width SiNW device. This finding is supported with the data obtained from the slope value of the linear regression in Figure S2 of Supplementary Materials. This is due to the increased surface area to volume ratio for smaller dimensions of NW, which has resulted in greater surface effects on the electrical conduction. The electrical characterization of the devices depended strongly on the width of the wires, which is in agreement with the idea that the influence from surface charges is enhanced when the width of the nanowire is decreased [33,34]. 

#### 3.3.2. Effect of Different Numbers of SiNW on Device Sensitivity

**Figure 6:**
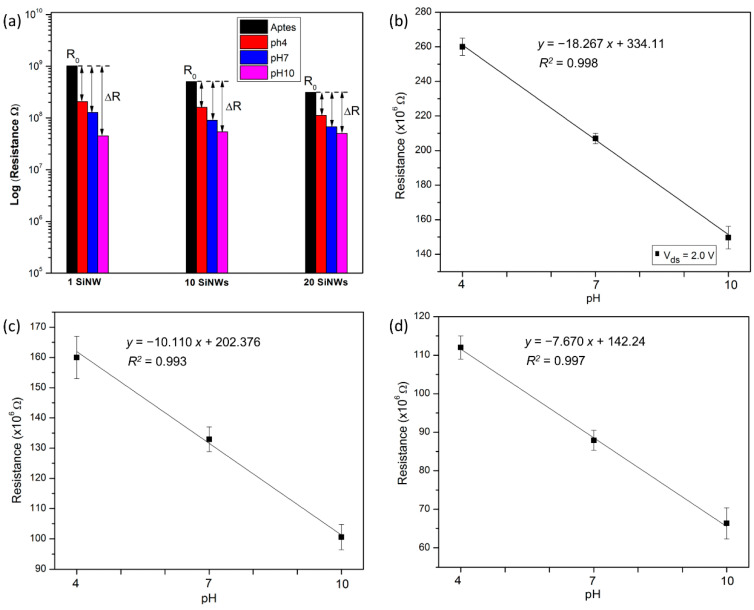
(**a**) The average resistance measurement of sensor devices with different numbers of SiNW (i.e., single NW, 10 NWs, and 20 NWs) for pH sensing. Change of electrical resistance by different pH levels for (**b**) single SiNW device, (**c**) 10 SiNWs device, and (**d**) 20 SiNWs device.

Previous studies suggested that the number of bridging nanowires incorporated into a device may be an important parameter in determining sensitivity [30,36]. To further explore this effect, single and multiple nanowires were fabricated as a sensing element. The multiple nanowires consist of an array of 10 NWs and 20 NWs in parallel. For this purpose, the width and length of NWs were fixed to 60 nm and 150 µm (sensing region), respectively, while the number of NWs was varied. As shown in Figure 6a, the device’s resistivity values are related to the number of Si wires. After performing surface modification using APTES, the average resistance for an APTES/SiNW device consisting of a single NW had the highest resistance measurement of 1013 × 10^6^ Ω, followed by the devices that consist of 10 NWs and 20 NWs with resistance values of 504 × 10^6^ Ω and 312 × 10^6^ Ω, respectively. These findings corroborate the results from previous studies [36,37,38], which suggests that a device that consists of multiple NWs in parallel allows more current flow as compared to the single NW. 

After introducing the pH solution, the average resistance for the device consisting of 20 NWs was 112 × 10^6^ Ω for pH 4, 88.7 × 10^6^ Ω for pH 7, and 67 × 10^6^ Ω for pH 10. A similar trend can be described for the device that consists of 10 NWs and a single NW, which exhibited the highest resistance at pH 4, followed by pH 7 and pH 10. It was demonstrated that the acid solution caused an increase in resistance, while the basic solution induced a decrease in resistance. These reactions were determined by the interaction of the surface silanol (SiOH) and amine (NH_2_) groups with the proton (H^+^) ions (Figure 5c). Moreover, the device that comprises a single SiNW yielded the maximum value of the resistance change (using Equation (1)), followed by an array of 10 SiNWs and 20 SiNWs, as presented in Table S2 of Supplementary Materials. The result is in agreement with previously reported work [32,36], suggesting that when using multiple SiNWs, the number of analyte-SiNW interactions for each SiNW is lower than if only a single SiNW were exposed to the same amount of analyte.

To additionally prove the sensitivity in pH response of amine-terminated SiNW with respect to the different numbers, the resistance values were plotted as a function of different pH levels, as shown in Figure 6b–d. The sensitivity of the SiNW can be determined by measuring the resistance change of the SiNW devices in response to the pH buffer solution change [39] and can be expressed as:

Sensitivity (S) = ∆Resistance/∆pH,(2)

The device that consists of a single NW showed the highest sensitivity of 18.26 × 10^6^ Ω/pH, which was obtained from the slope value of the linear regression (*y* = m*x* + *c*) in Figure 6b, followed by 10.11 × 10^6^ Ω/pH for 10 SiNWs (Figure 6c) and 7.67 × 10^6^ Ω/pH for 20 SiNWs (Figure 6d). In addition, the results confirm that the pH dependence was linear for all the samples in the pH range from 4 to 10. The satisfactory result obtained indicates the efficiency of the protocol applied for SiNWs surface modification using APTES in this research. This is based on its effective function as a sensitive detector for sensing pH and, thus, could be employed for further stepwise ssDNA surface functionalization.

### 3.4. DNA Hybridization Detection

**Figure 7:**
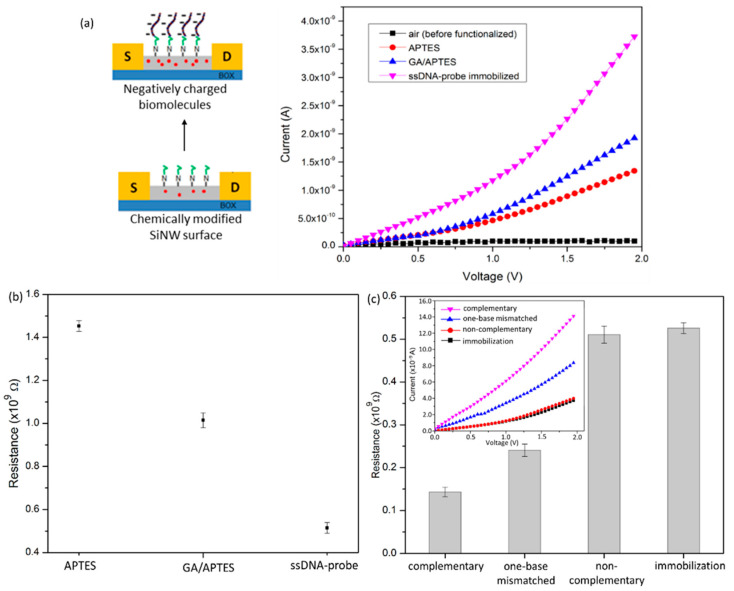
(**a**) Schematic illustration of p-type SiNW detection scheme (**left**) and electrical measurement of the different step-functionalized SiNW sensors (**right**). (**b**) The resistance measurement at V_ds_ = 2V of the fabricated device is plotted with respect to the different stages of surface functionalization. (**c**) Selectivity studies of developed ssDNA-based SiNW sensors towards different target DNA (complementary, one-base mismatched, and non-complementary).

The devices composed of a single NW with 60 nm in width were further used in the detection of biomolecules. Figure 7a illustrates the working principle of the SiNW-based DNA biosensor. In this configuration, the binding of biomolecules that have a net-negative charge to the SiNW surface leads to the accumulation of charge carriers (holes) in the p-type SiNW channel [32]. This reaction can be detected simply as a decrease in the resistance of the device, as experimentally verified using a picoamperometer-based electrical detection. 

A significant change in the resistance of the device was observed for bare electrical testing and modified SiNW using APTES. The device showed a detectable current value of 1.46 × 10^−9^ A, in response to the chemically modified with APTES (Figure 7a). It was found that chemical passivation of the NW surfaces using different chemical functional groups (such as –NH_2_) reduced the band gap of the NW, thus allowing for electrical conductivity. This phenomenon was supported by Byon [40], who claimed that the intrinsic dipole moments of different kinds of linker molecules affect the semiconductor band gap upon their interaction with semiconductor surfaces, can be used to tune electrical properties controllable, and can also be used to overcome doping difficulties for sensors with thin layers in an active region. Then, the current measurement for a GA/APTES surface modification was increased to 2.0 × 10^−9^ A, pointing to a 40% change of resistance. It was found that the surface termination by aldehyde groups generated a layer of negative surface charge [41], thus accumulating hole carrier in the p-type SiNW surface and resulting in increased current. After the introduction of 1.0 × 10^−6^ M of probe ssDNA to the GA/APTES modified SiNW surface, the ssDNA immobilization causes a further increment in current flow. The obtained signals are in agreement with the illustration of theoretical predictions, as shown in Figure 7a (left), implying that the attachment of negatively charged ssDNA-probe caused the accumulation of charge carrier (holes) in the p-type SiNW and resulting in the decrease in electrical resistance (Figure 7b). 

The ssDNA-functionalized SiNWs were further evaluated with 1.0 × 10^−6^ M of complementary DNA, 1.0 × 10^−6^ M of one-base mismatch DNA, and 1.0 × 10^−6^ M of non-complementary DNA, respectively for the specificity of the fabricated device as a sensor. The insert of Figure 7c shows the hybridization with fully complementary target DNA caused the highest increment in current flow, since more negative charges are trapped on the surface. The resistance measurement of the device before and after hybridization is approximately 0.52 × 10^9^ Ω and 0.14 × 10^9^ Ω, respectively, which corresponds to a 73% signal change. This observation coincides well with previously reported works [5,42], suggesting that the build-up of negative charges on the nucleic acid phosphate backbones after the DNA hybridization at the SiNW surface causes the accumulation of charge carrier (holes) in the p-type SiNWs sensor and results in decreased resistance. A control experiment with a non-complementary target showed no change in the current, indicating the absence of nonspecific target binding. 

Furthermore, our fabricated devices could specifically discriminate between matched and mismatched DNA sequences, by responding to 50% resistance change toward one-base mismatched target DNA in comparison with a complementary target DNA–ssDNA-probe binding. The weakly attached one-base mismatched target DNA is removed during the washing process due to poor or incomplete hybridization of the target and resultant decrease in the current measurement. In this study, the hybridization of the target DNA was easily confirmed by the observation of the current changing of ssDA-functionalized SiNW sensor in the presence of targets with the following order, “complementary > one-base mismatch > non-complementary”, without introducing an indicator or other label molecules. 

**Figure 8:**
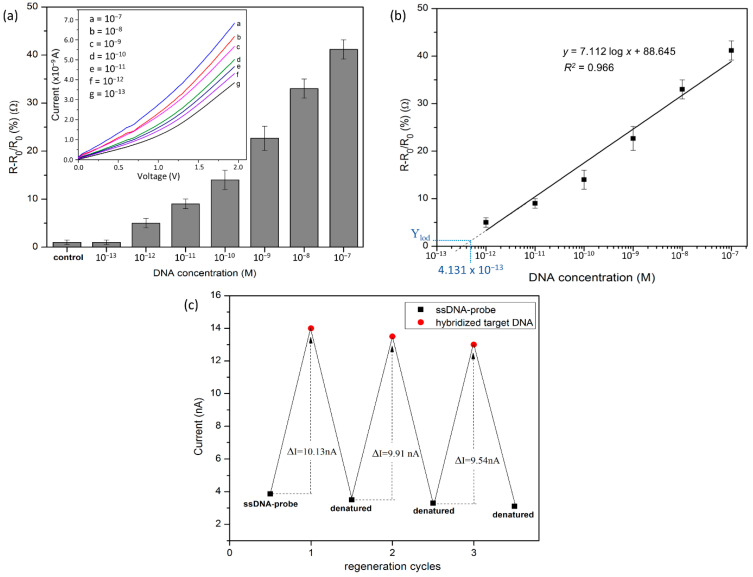
(**a**) The detection response of the fabricated SiNW device towards different concentrations of fully complementary target DNA. (**b**) The calibration curve of the resistance changes corresponding to the target DNA concentration range of 1.0 × 10^−12^ M to 1.0 × 10^−7^ M. (**c**) The regeneration of ssDNA-functionalized SiNW.

The sensor device was further evaluated for sensitivity detection by monitoring the resistance change caused by the binding of the hybridized target DNA to immobilize the probe DNA. The detection response of the devices can be defined as the relative change in resistance (∆R/R_0_), where R_0_ is the initial resistance of immobilized probe DNA, and ∆R is the change in resistance after hybridizing with the target DNA [43]. As depicted in Figure 8, the resistance of the SiNW sensors increased linearly (*R*^2^ = 0.966), with the complementary target DNA concentration in the range of 1.0 × 10^−7^ M to 1.0 × 10^−12^ M. It is known that the change in surface charges after hybridization is associated with the target concentrations, with more charges being present as the target concentration is increased [43]. Barely any resistance change was observed for the further dilution of DNA concentration down to 1.0 × 10^−13^ M, which is similar to the control signal (non-complementary target DNA). In addition to sensitivity, the limit of detection (LoD) of the SiNW sensors can be calculated from the following equation:

Y_LoD_ = Y_blank_ + 3SD_blank_, (3)

where Y_blank_ is the mean of the blank measurement, and SD_blank_ is the standard deviation of the blank measurement. The value of the non-complementary target DNA measurement is used as a blank measurement value. The linear-regression equation of the plots is *y* = 7.112 log*x* + 88.645, where x is the concentration of target DNA, and y is the calculated Y_lod_ value from Equation (3). The correlation coefficient of 0.966 demonstrated the reliability of the biosensor with the associated concentration for an LoD of 4.131 × 10^−13^ M. The developed sensor shows a competitive detection limit and a wide detection range compared to conventional electrochemical-based sensors, as shown in Table 2. SiNW biosensors possess several merits as field-effect devices [44,45] that make them suitable for ultrasensitive biosensing, without the need for labels or signal amplification steps. Furthermore, the short incubation time, simplicity, and rapid detection make SiNW biosensors a viable alternative to the traditional enzyme-linked immunosorbent assay (ELISA) [46] in the detection of DNA hybridization events.

Furthermore, the fabricated SiNW sensor shows reversibility for three cycles of hybridized DNA (double-stranded DNA) and denatured DNA (single-stranded DNA immobilization), with the current response repeatedly between 9.5 × 10^−9^ A and 10.2 × 10^−9^ A performed with the same device (Figure 8c). Denaturation was performed by washing the device with hot DI water (~85 °C) to separate the hybridized target DNA from the probe surface [52]. As confirmed again by measuring the I–V relations, the curve returned to almost the same level as the immobilization curve, indicates that the denaturation was fully done. The developed sensor demonstrates analytical reliability with a relative standard deviation (R.S.D) of 4.1% after three cycles, showing that the functionalized SiNW-based ssDNA can be used as a reusable biosensor. Furthermore, the sensor shows an acceptable detection stability, as the signal retained 90.5% of its initial current after five days of storage at 4 °C.

## 4. Conclusions

We have designed SiNW sensors via a top-down fabrication approach, using EBL coupled with conventional photolithography. A device with different numbers of NWs (i.e., single, 10, and 20 NWs) with varied widths was successfully fabricated on the SOI substrate. The device performance was tested in the detection of different level pH solutions. It was found that amine functionalized SiNW surface undergoes protonation and deprotonation, yielding promising results as a pH sensor. The most sensitive pH response of 18.26 × 10^6^ Ω/pH was observed for the sensor consisting of a single NW of 60 nm width. The sensor platform was extended for DNA hybridization detection, by introducing a bifunctional linker glutaraldehyde, a cross-linker providing the covalent attachment of NH_2_-terminated ssDNA probe via its aldehyde group. A reliable detection of dengue virus (DENV) DNA oligomer for non-complementary, one-base mismatched, and fully complementary base pairs was observed, based on the changes in the electrical resistivity of the ssDNA-modified SiNW channel devices. The developed sensors demonstrated a low detection limit with a wide linear detection range. Thus, the flexibility of SiNW as a sensing platform can be further used with a wide range of bio-receptor molecules for the diagnosis of a variety of diseases.

## Figures and Tables

**Table 1:** C1s spectra of Si surface modified with APTES and glutaraldehyde.

Species	C1 (C–C, C–H) % Binding Energy (eV)	C2 (C–N, C–O) % Binding Energy (eV)	C3 (C=O, O–C–O) % Binding Energy (eV)
APTES	87.96 (285.05)	12.04 (286.95)	-
Glutaraldehyde	80.28 (285.16)	15.09 (286.65)	4.64 (288.05)

**Table 2:** Analytical performance of DNA biosensors.

Assay	Device Specification	Detection Range	Detection Limit	Ref.
Electrochemical	SiNW/AuNPs/SPGE	0.01–50 nM	0.01 nM	[47]
	rGO/PGE	0.05–200 µM	0.023 µM	[48]
	Gr/ppy/SPCE	1.0 µM–1.0 nM	0.78 µM	[49]
Electrical	n-type SiNW (w = 10 nm)	100 pM–25 nM	0.1 nM	[5]
	p-type SiNW (w = 40 nm)	100–1000 pM	200 pM	[50]
	p-type SiNW (w = 20 nm)	10 nM–0.1 fM	0.1 fM	[51]
	p-type SiNW (w = 60 nm)	1.0 pM–0.1 µM	0.4 pM	This work

Note: SPGE: screen-printed gold electrode, rGO: reduced graphene oxide, PGE: pencil graphite electrode, Gr: graphene, ppy: polypyrrole, SPCE: screen printed carbon electrode, SiNW: silicon nanowire, w: width.

## Data Availability

The data presented in this study are available on request from the corresponding author.

## Supplementary Materials

The following are available online at https://www.mdpi.com/article/10.3390/nano12152652/s1, Figure S1: FESEM images of SiNWs with diameter of approximately (a) 60 nm, (b) 70 nm, (c) 80 nm, (d) 90 nm and (e) 100 nm at 100 KX magnification; Figure S2: The devices consist of SiNW with (a) 60 nm width, (b) 70 nm width, (c) 80 nm width, (d) 90 nm width and (e) 100 nm width shows the highest resistance value at pH 4 and lowest resistance value at pH 10 with linear relationship between pH level and resistance measurement at 2V, respectively; Table S1: Effect of SiNW width on detection response, Table S2: Effect of SiNWs number on pH detection response.
