# Supplementary material for: Fabrication of Silicon Nanowire Sensors for Highly Sensitive pH and DNA Hybridization Detection
Source: Nanomaterials (Basel). 2022 Aug 2;12(15):2652. doi: 10.3390/nano12152652 (PMC9370444; doi:10.3390/nano12152652)
Supplement: Supplementary file 1 [file nanomaterials-12-02652-s001.zip › nanomaterials-1823857-supplementary.pdf]

nanomaterials

MDPI

Supplementary Materials

Fabrication of Silicon Nanowire Sensors for Highly Sensitive pH and DNA Hybridization Detection

Siti Fatimah Abd Rahman [1,*], Nor Azah Yusof [2,3,*], Mohd Khairuddin Md Arshad [1,4], Uda Hashim [1], Mohammad Nuzaihan Md Nor [1] and Mohd Nizar Hamidon [2]

[1] Institute of Nano Electronic Engineering, Universiti Malaysia Perlis, Kangar 01000, Perlis, Malaysia; mohd.khairuddin@unimap.edu.my (M.K.M.A.); uda@unimap.edu.my (U.H.); m.nuzaihan@unimap.edu.my (M.N.M.N.)
[2] Institute of Nanoscience and Nanotechnology, Universiti Putra Malaysia, Serdang 43400, Selangor, Malaysia; mnh@upm.edu.my (M.N.H)
[3] Department of Chemistry, Faculty of Science, Universiti Putra Malaysia, Serdang 43400, Selangor, Malaysia
[4] Faculty of Electronic Engineering Technology, Universiti Malaysia Perlis, Arau 02600, Perlis, Malaysia
* Correspondence: siti_fatimah0410@yahoo.com (S.F.A.R.); azahy@upm.edu.my (N.A.Y.)

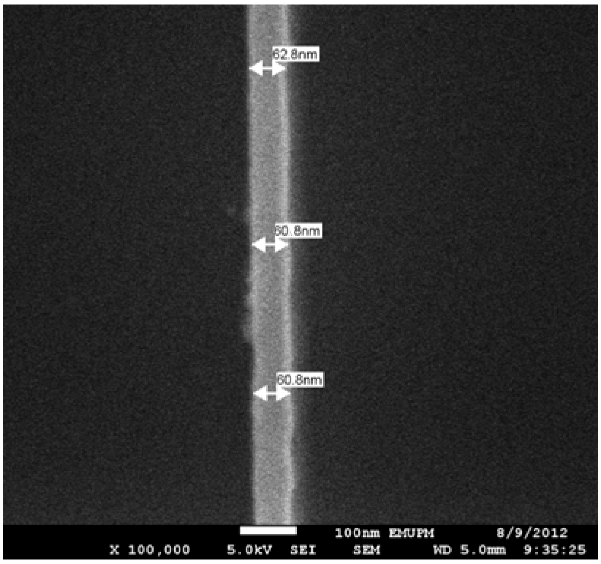

(**a**)

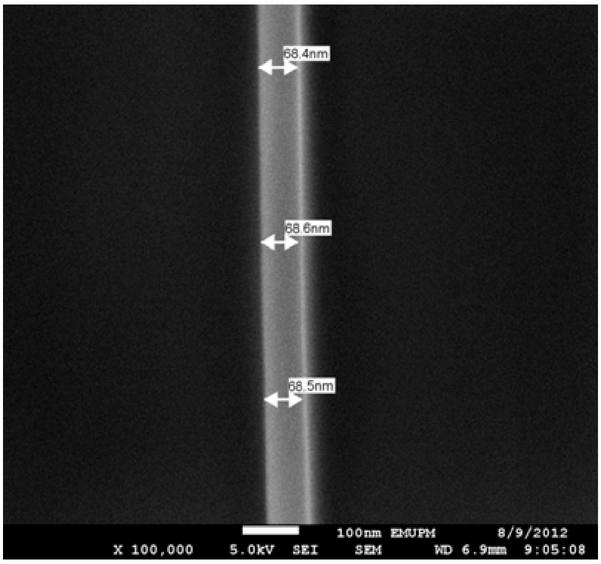

(**b**)

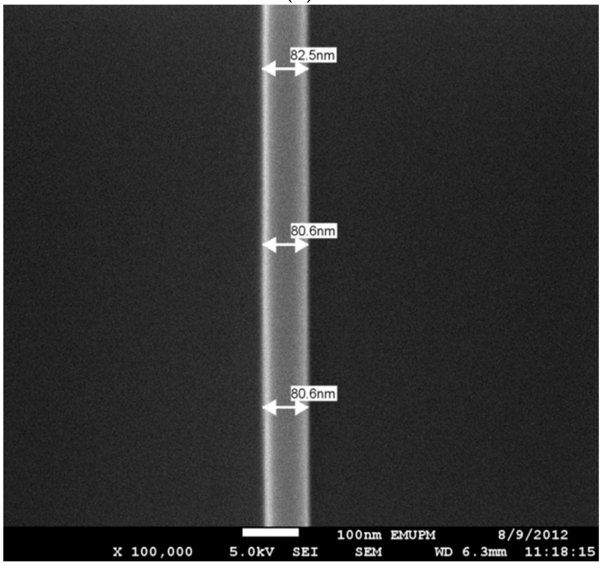

(**c**)

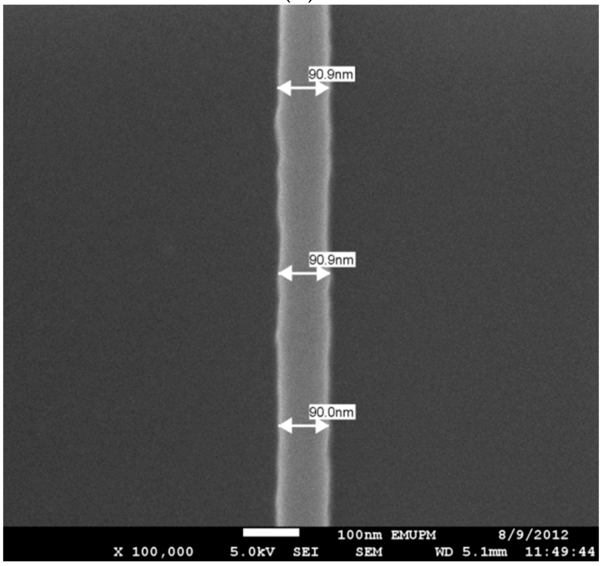

(**d**)

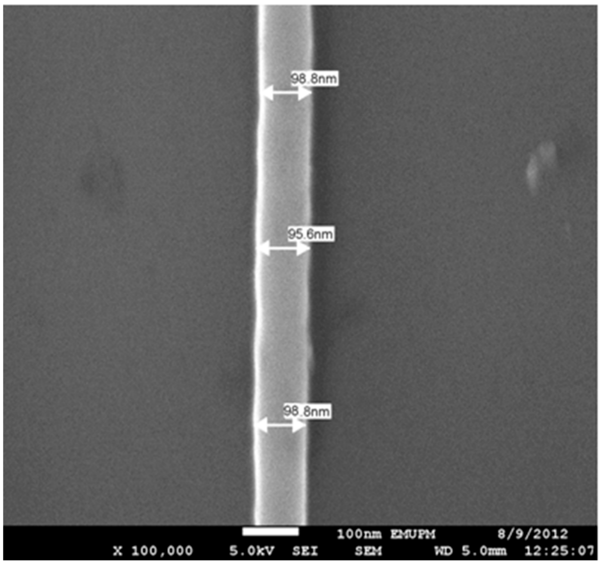

(**e**)

Figure S1. FESEM images of SiNWs with diameter of approximately (**a**) 60 nm, (**b**) 70 nm, (**c**) 80 nm, (**d**) 90 nm and (**e**) 100 nm at 100 KX magnification.

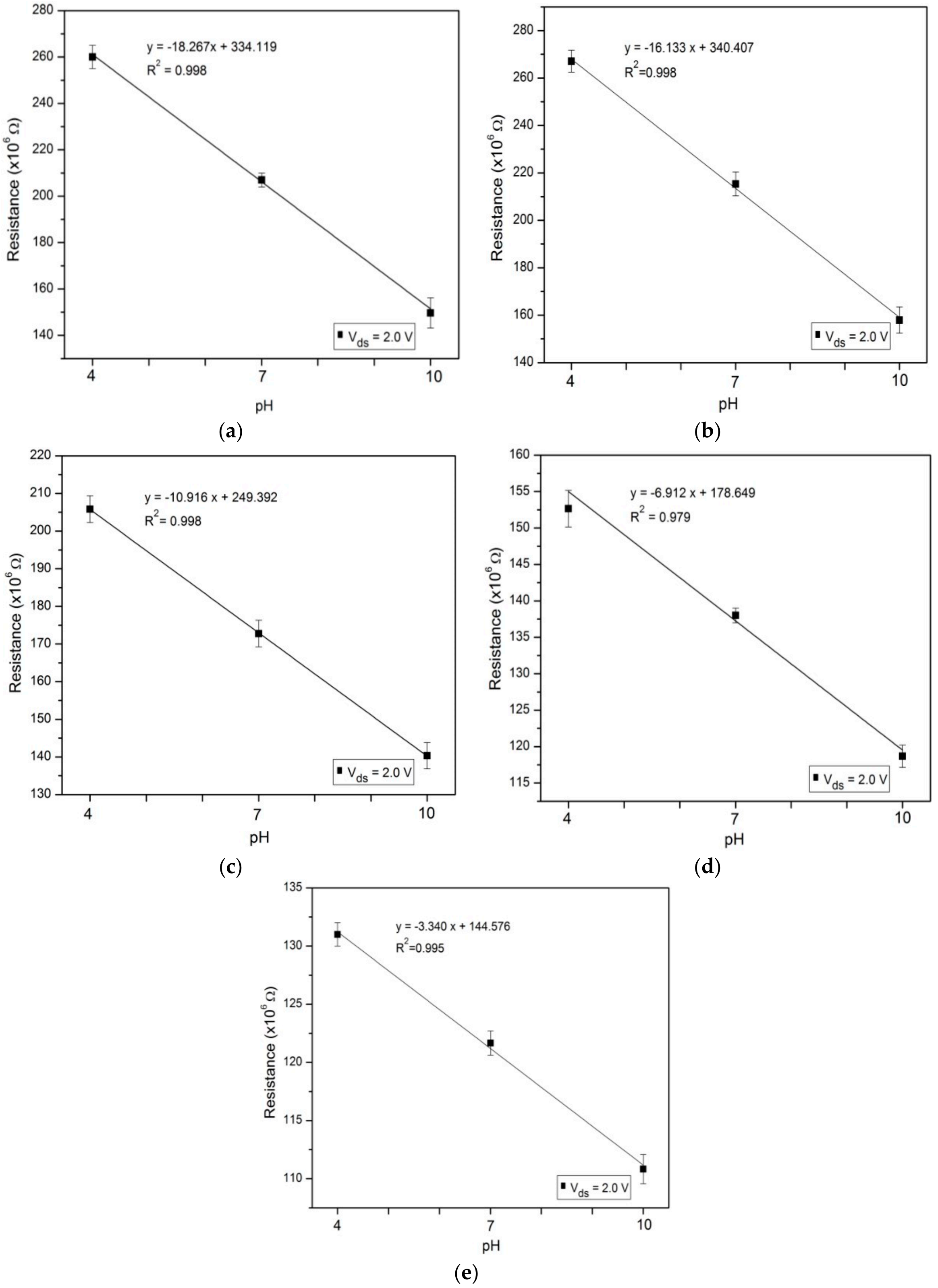

Figure S2. The devices consist of SiNW with (**a**) 60 nm width, (**b**) 70 nm width, (**c**) 80 nm width, (**d**) 90 nm width and (**e**) 100 nm width shows the highest resistance value at pH 4 and lowest resistance value at pH 10 with linear relationship between pH level and resistance measurement at 2V, respectively.

Table S1. Effect of SiNW width on detection response.

pH	SiNW width (nm)	Average resistance (MΩ)		ΔR/R0 (%)
		R0 (APTES)	ΔR	
4	60	1013.0	753.0	74.3
	70	725.0	456.7	63.0
	80	450.0	243.4	54.0
	90	255.0	102.5	40.1
	100	172.0	40.3	23.4
7	60	1013.0	806.2	79.6
	70	725.0	508.8	70.2
	80	450.0	276.8	61.5
	90	255.0	118.0	46.3
	100	172.0	50.5	30.0
10	60	1013.0	863.2	85.2
	70	725.0	566.2	78.0
	80	450.0	310.0	69.0
	90	255.0	136.0	53.3
	100	172.0	62.0	36.0

Table S2. Effect of SiNWs number on pH detection response.

pH	SiNW number	Average resistance (MΩ)		S=ΔR/R0 (%)
		R0 (APTES)	ΔR	
4	1	1013.0	753.0	74.3
	10	504.0	344.0	68.3
	20	312.0	200.0	64.1
7	1	1013.0	806.2	79.6
	10	504.0	371.2	73.7
	20	312.0	223.3	71.6
10	1	1013.0	863.2	85.2
	10	504.0	402.2	79.8
	20	312.0	245.0	78.5
